# Analytical Solution of the Time-Dependent Microfluidic Poiseuille Flow in Rectangular Channel Cross-Sections and Its Numerical Implementation in Microsoft Excel

**DOI:** 10.3390/bios9020067

**Published:** 2019-05-24

**Authors:** Patrick Risch, Dorothea Helmer, Frederik Kotz, Bastian E. Rapp

**Affiliations:** Department of Microsystems Engineering (IMTEK), Laboratory of Process Technology | NeptunLab, Albert-Ludwigs University of Freiburg, 79085 Freiburg im Breisgau, Germany; Patrick.Risch@imtek.de (P.R.); Dorothea.Helmer@imtek.de (D.H.); Frederik.Kotz@imtek.de (F.K.)

**Keywords:** microfluidics, numerical techniques, Microsoft Excel, finite difference method, Navier–Stokes, time dependent flow, dynamic flow, initiation of flow, Poiseuille flow

## Abstract

We recently demonstrated that the Navier–Stokes equation for pressure-driven laminar (Poiseuille) flow can be solved in any channel cross-section using a finite difference scheme implemented in a spreadsheet analysis tool such as Microsoft Excel. We also showed that implementing different boundary conditions (slip, no-slip) is straight-forward. The results obtained in such calculations only deviated by a few percent from the (exact) analytical solution. In this paper we demonstrate that these approaches extend to cases where time-dependency is of importance, e.g., during initiation or after removal of the driving pressure. As will be shown, the developed spread-sheet can be used conveniently for almost any cross-section for which analytical solutions are close-to-impossible to obtain. We believe that providing researchers with convenient tools to derive solutions to complex flow problems in a fast and intuitive way will significantly enhance the understanding of the flow conditions as well as mass and heat transfer kinetics in microfluidic systems.

## 1. Introduction

The fundamental physics of flows in microchannels are pivotal for the precise control of dynamic effects underlying transport phenomena such as momentum, mass or heat transfer and ultimately define the behavior or the system at hand for a practical application. As an example, establishing the correct flow physics is crucial for modeling and understanding the transport kinetics of an analyte from the bulk of the flow to the surface of, e.g., a biosensor [1]. In particular, the response dynamics of a biosensor strongly depends on the diffusion kinetics of the analyte within the bulk of the flow of the biosensor. The formation of the Nernst diffusion layer is the limiting factor that defines the transport dynamics and thus the dynamic response of the biosensor [2]. This is particularly important during the initiation of the flow and in transitions of the flow such as, e.g., at the early stage of an experiment. These are tightly interlinked with the fluid mechanics of the system and thus a detailed understanding, specifically of transient effects in the bulk fluid flow, is pivotal for a correct system assessment [3]. In many respects, the fluid physics of microfluidics are straight-forward. Given the low Reynolds number flows commonly encounter in microfluidics, effects such as turbulence are rarely relevant in microfluidic flows [4]. 

However, given the fact that the underlying equation of fluid dynamics, i.e., the Navier–Stokes equation, is very difficult to solve even in (seemingly) simple channel geometries and flow conditions, researchers commonly refer to numerical methods to model the flow physics [5]. However, correctly applying a complex numerical modeling or solver software package is usually beyond the scope of application-driven research in microfluidics and adjacent fields.

We have recently demonstrated, that the simplified Navier–Stokes equation for laminar flow can be conveniently solved in a spreadsheet analysis software such as Microsoft Excel [6,7]. Spreadsheet tools are convenient platforms for implementing schemes in an intuitive and documentable manner. Software packages such as Microsoft Excel are widely available, and researchers are used to working with these packages from their daily work routine. Thus, applying a scheme which is implemented on this platform comes with a significantly lower barrier than adopting a complex and costly numerical solver package.

In our two most recent contributions, we demonstrated that the Poiseuille equation, a simplified version of the Navier–Stokes equation for stationary laminar flows, can be implemented in a wide range of channel geometries. For geometries for which analytical solutions can be obtained, the numerical results from the spreadsheet deviate only by a few percent from the analytical solutions [6]. We also demonstrated that the spreadsheet can be used to implement different boundary conditions besides the commonly employed no-slip (Dirichlet) boundary condition such as, e.g., Neumann-type boundary conditions which occur on slip surfaces as well as on open channel geometries [7]. These solutions are close-to-exact to the analytical solutions, which again, can only be derived for very simple channel geometries, usually with a high degree of symmetry such as, e.g., in circular channel (Hagen–Poiseuille) flows.

However, all of these solutions were derived for stationary flow scenarios, i.e., flows for which the time-dependency of the Poiseuille equation is ignored. This case is applicable for applications where the flow is assumed to be in steady-state and flow acceleration (i.e., during initiation of the flow by applying a pressure drop), as well as flow retardation (i.e., during stopping of the flow by removing the driving pressure drop) are not taken into account. However, there are many applications where time-dependence needs to be considered, especially in applications where the momentum diffusion is overlaid by, e.g., mass diffusion. In this paper we will show that the spreadsheets can be designed to reflect time dependency, allowing the study of transient effects during flow initiation and retardation, as well as intermediate changes in the driving pressure drop which modifies the flow conditions.

## 2. Numerical Scheme

### 2.1. Navier–Stokes Equation for Time-Dependent Flow

In comparison to the stationary version of the Navier–Stokes equation used in [6], we now have to consider time-dependency. Neglecting volume forces, the Navier–Stokes equation simplifies to

(1) $$\rho \frac{\partial \vec{v}}{\partial t}=-\vec{\nabla }p+\eta \Delta \vec{v}$$

which includes pressure contribution ($\vec{\nabla }p)$, momentum diffusion ($\eta \Delta \vec{v})$, as well as time-dependency ($\frac{\partial \vec{v}}{\partial t}$). Again, we consider parallel flow which reduces the dependent variable $\vec{v}$ (a vector) to only the contribution $v_{x}$ along the x-axis. Equation (1) can therefore be written as

(2) $$\rho \frac{\partial v_{x}}{\partial t}=-\frac{\Delta p}{\Delta L}+\eta \left(\frac{\partial^{2}v_{x}}{\partial y^{2}}+\frac{\partial^{2}v_{x}}{\partial z^{2}}\right)$$

where we also note that the only driving pressure drop is the drop along the x-axis of the channel given by $\frac{\Delta p}{\Delta L}$.

### 2.2. Numerical Scheme for the Second-Order Partial Differential Equations

In [6] we derived numerical schemes for second-order partial differentials from the Taylor series given by

(3) $$\frac{d^{2}f}{dx^{2}}\left(x_{0}\right)=\frac{f\left(x_{0}+\Delta x\right)+f\left(x_{0}-\Delta x\right)-2f\left(x_{0}\right)}{{\left(\Delta x\right)}^{2}}$$

which allowed us to rewrite the right-hand side of Equation (2) to

(4) $$\begin{array}{c} \rho \frac{\partial v_{x}}{\partial t}=-\frac{\Delta p}{\Delta L}+\\ \eta \left(\frac{v_{x}\left(y_{0}+\Delta y,z_{0}\right)+v_{x}\left(y_{0}-\Delta y,z_{0}\right)-2v_{x}\left(y_{0},z_{0}\right)}{{\left(\Delta y\right)}^{2}}+\frac{v_{x}\left(y_{0},z_{0}+\Delta z\right)+v_{x}\left(y_{0},z_{0}-\Delta z\right)-2v_{x}\left(y_{0},z_{0}\right)}{{\left(\Delta z\right)}^{2}}\right) \end{array}$$

which we can further simplify by using a common step width in space $h_{yz}=\Delta y=\Delta z$ to yield

(5) $$\begin{array}{c} \rho \frac{\partial v_{x}}{\partial t}=-\frac{\Delta p}{\Delta L}+\\ \eta \left(\frac{v_{x}\left(y_{0}+h_{yz},z_{0}\right)+v_{x}\left(y_{0}-h_{yz},z_{0}\right)+v_{x}\left(y_{0},z_{0}+h_{yz}\right)+v_{x}\left(y_{0},z_{0}-h_{yz}\right)-4v_{x}\left(y_{0},z_{0}\right)}{h_{yz}^{2}}\right) \end{array}$$

This scheme uses a so-called finite difference scheme to approximate the second order partial differential by considering the changes in the function over a finite difference in the independent variables y and z. This scheme is a second-order scheme and thus numerically very stable.

Introducing the general notation $F^{\left(t,y,z\right)}$ for the value of $v_{x}$ at the position $\left(y_{0},z_{0}\right)$ at time point t, $F^{\left(t,y+1,z\right)}$ for the value of $v_{x}$ at the position $\left(y_{0}+h_{yz},z_{0}\right)$ at time point t, $F^{\left(t,y-1,z\right)}$ for the value of $v_{x}$ at the position $\left(y_{0}-h_{yz},z_{0}\right)$ at time point t, $F^{\left(t,y,z+1\right)}$ for the value of $v_{x}$ at the position $\left(y_{0},z_{0}+h_{yz}\right)$ at time point t and $F^{\left(t,y,z-1\right)}$ for the value of $v_{x}$ at the position $\left(y_{0},z_{0}-h_{yz}\right)$ at time point t we can rewrite Equation (5) to

$$\frac{\partial v_{x}}{\partial t}=-\frac{\Delta p}{\Delta L}+\eta \left(\frac{F^{\left(t,y+1,z\right)}+F^{\left(t,y-1,z\right)}+F^{\left(t,y,z+1\right)}+F^{\left(t,y,z-1\right)}-4F^{\left(t,y,z\right)}}{h_{yz}^{2}}\right)$$

(6) $$\frac{\partial v_{x}}{\partial t}=-\frac{1}{\rho }\frac{\Delta p}{\Delta L}+\frac{\eta }{\rho }\left(\frac{F^{\left(t,y+1,z\right)}+F^{\left(t,y-1,z\right)}+F^{\left(t,y,z+1\right)}+F^{\left(t,y,z-1\right)}-4F^{\left(t,y,z\right)}}{h_{yz}^{2}}\right)$$

### 2.3. Numerical Scheme for the First-Order Partial Differential with Respect to Time

The second partial differential required for setting up the numerical scheme is the first-order partial differential with respect to time. For this we expand the Fourier series in the positive direction given by

(7) $$f\left(x_{0}+\Delta x\right)=\overset{n_{\max}}{\underset{n=0}{\sum }}\frac{1}{n!}{\frac{d^{n}f}{dx^{n}}|}_{x_{0}}\Delta x^{n}+O_{n_{\max}+1}$$

to $n_{\max}=1$, where $n_{\max}$ is the “expansion order” and $O_{n_{\max}}$ is the error function of order $n_{\max}$. We subsequently obtain

$$f\left(x_{0}+\Delta x\right)=f\left(x_{0}\right)+\frac{df}{dx}\Delta x+O_{2}$$

(8) $$\frac{df}{dx}=\frac{f\left(x_{0}+\Delta x\right)-f\left(x_{0}\right)}{\Delta x}$$

which is a first-order approximation for the first derivative in time (a so-called forward Euler scheme). Combining Equations (8) and (6) results in the numerical scheme

$$\frac{F^{\left(t+1,y,z\right)}-F^{\left(t,y,z\right)}}{h_{t}}=-\frac{1}{\rho }\frac{\Delta p}{\Delta L}+\frac{\eta }{\rho }\left(\frac{F^{\left(t,y+1,z\right)}+F^{\left(t,y-1,z\right)}+F^{\left(t,y,z+1\right)}+F^{\left(t,y,z-1\right)}-4F^{\left(t,y,z\right)}}{h_{yz}^{2}}\right)$$

$$F^{\left(t+1,y,z\right)}=F^{\left(t,y,z\right)}-\frac{h_{t}}{\rho }\frac{\Delta p}{\Delta L}+\frac{h_{t}\cdot \eta }{\rho }\left(\frac{F^{\left(t,y+1,z\right)}+F^{\left(t,y-1,z\right)}+F^{\left(t,y,z+1\right)}+F^{\left(t,y,z-1\right)}-4F^{\left(t,y,z\right)}}{h_{yz}^{2}}\right)$$

$$F^{\left(t+1,y,z\right)}=F^{\left(t,y,z\right)}\left(1-4\frac{h_{t}\cdot \eta }{\rho h_{yz}^{2}}\right)+\frac{h_{t}\cdot \eta }{\rho }\left(\frac{F^{\left(t,y+1,z\right)}+F^{\left(t,y-1,z\right)}+F^{\left(t,y,z+1\right)}+F^{\left(t,y,z-1\right)}}{h_{yz}^{2}}\right)-\frac{h_{t}}{\rho }\frac{\Delta p}{\Delta L}$$

(9) $$F^{\left(t+1,y,z\right)}=F^{\left(t,y,z\right)}\left(1-4\Omega \right)+\Omega \left(F^{\left(t,y+1,z\right)}+F^{\left(t,y-1,z\right)}+F^{\left(t,y,z+1\right)}+F^{\left(t,y,z-1\right)}\right)-\Gamma$$

with $\Omega =\frac{h_{t}\Delta \eta }{\rho h_{yz}^{2}}$ and $\Gamma =\frac{h_{t}}{\rho }\frac{\Delta p}{\Delta L}$, $h_{t}$ being the step width in time. Equation (9) is a second-order scheme with respect to space and a first-order scheme with respect to time. It allows stepping forward in time from a known value $F^{\left(t,y,z\right)}$ at the location (x,y) at time t to the unknown value $F^{\left(t+1,y,z\right)}$ at the location (x,y) at time t+1. Compared to the schemes used in [6] this scheme is not iterative as the solution at a given time point t does not have to fulfill the Navier–Stokes equation but only Equation (8). One point of note is the fact that the numerical scheme in time is only first-order. In order to not risk numerical instability the step width $h_{t}$ in time must be chosen sufficiently small. In general, first-order approximations are numerically significantly less stable than higher-order implementations but they are computationally very cost-effective and thus very simple to implement. The numerical stability of the overall scheme hinges mostly on the step width in time.

### 2.4. Correcting Units

Before implementing the numerical scheme we must correct for the units. We assume $h_{xy}$ to be given in μm, $\frac{\Delta p}{\Delta L}$ to be given in mbar/mm, ρ to be given in g/cm^3^, η to be given in mPa·s and $h_{t}$ to be given in μs. The unit of the dependent variable $v_{x}$ is mm/s and the independent variables y and z are given in μm. In order to correct for the units, Γ has a prefactor of 0.1, whereas Ω has a prefactor of 1.

## 3. Implementation in Microsoft Excel

### 3.1. Layout of the Spreadsheet

**Figure 1:**
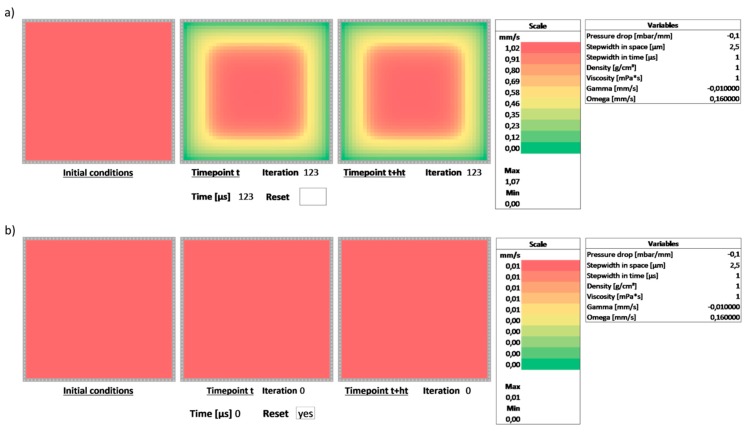
View of the Microsoft Excel spreadsheets with the three panels: initial conditions (left), current time point (center) and next time point (right). (**a**) The evolution of the velocity profile can be observed by pressing the F9 key. The right panel implements Equation (9) and steps forward in time. The values are copied back to the center panel thus performing one iteration. (**b**) By adding any value into the “Reset” field, the scheme is reset, the iteration counter is cleared and the values of the initial condition (left panel) is copied into the center panel thus setting the velocity profile for time point t=0.

The scheme given by Equation (9) was implemented in Microsoft Excel in a spreadsheet, which can be downloaded from the supporting material (file “TimedependentMicrofluidicFlows.xlsx”). It is shown in Figure 1. The numerical domain was chosen as a 40 × 40 cell grid panel with no-slip boundary conditions. As demonstrated in an earlier contribution, different boundary conditions can be implemented such as, e.g., flip or Neumann-type boundary conditions [7]. The values in the cells represent the velocity of the flow at the given position in the domain. The sheet consists of three panels:

- left panel—initial conditions: these are the values of the flow in the channel at the beginning of the calculation; for a first demonstration, we assume the flow to be non-moving, i.e., all values are 0
- center panel—velocity profile at time point t: this is the velocity profile in the channel at the current timepoint, i.e., $F^{\left(t,y,z\right)}$; the scheme is assumed to step from this point to $F^{\left(t+1,y,z\right)}$
- right panel—velocity profile at time point $t+h_{t}$: this is the velocity profile calculated by stepping from timepoint t via the numerical scheme of Equation (9)

The panels are color-coded to reflect areas of higher velocity in red and areas of lower velocity in green. Next to the right-most panel, the color scale for the velocity profile for the right-most panel, i.e., $F^{\left(t+1,y,z\right)}$ is displayed. Below the color scale is the section for the variables. These values can be changed to modify, i.e., the type of fluid or the properties of the numerical scheme. The numerical scheme is corrected for the following units:

- independent variables y and z: μm
- pressure drop $\frac{\Delta p}{\Delta L}$: mbar/mm
- step width in space $h_{yz}$: μm
- step width in time $h_{t}$: μs
- density of the fluid: g/cm^3^
- viscosity of the fluid: mPa·s

Changing the value of the step width in space effectively increases the lateral dimension of the channel. Changing the value of the step width in time increases the speed of the calculation by assuming larger steps in the forward Euler scheme. However, as discussed, increasing this value may lead to the numerical scheme becoming unstable. This can be observed by the values of the velocity increasing continually until they overflow. Below the adjustable variables are the two variables used as an abbreviation in Equation (9), i.e., Ω and Γ which are updated dynamically.

### 3.2. Iteration

As discussed, the numerical scheme of Equation (9) does not require iteration within one time point. This is in contrast to the schemes implemented in [6], which require the steady-state version of the Poiseuille Equation (2) to be fulfilled for each position within the domain. Here, the scheme is required to perform one step in time but not to iterate further. 

However, due to the nature of the scheme, we require circular references in the spreadsheet, which means we have to allow iteration. In Microsoft Excel, select “File→Options→Formulas→Calculation options” and check “Enable iterative calculation”. Set “Maximum Iterations” to 1. This ensures that the scheme will only perform one single iteration.

### 3.3. Implementation of the Numerical Scheme

The scheme is implemented in the spreadsheet by taking the initial value from the left panel and copying the values into the center panel at the beginning of the calculation. The scheme then uses these values to calculate $F^{\left(t+1,y,z\right)}$ in the right panel from the values $F^{\left(t,y,z\right)}$ of the center panel. For the next step in time, the value of the right panel is copied back into the respective cell of the center panel. This is the single iteration which Microsoft Excel will perform—the value will not be updated further. The formulae of the cells in the right panel implement the numerical scheme. They use the value $F^{\left(t,y,z\right)}$ as well as $F^{\left(t,y+1,z\right)}$, $F^{\left(t,y-1,z\right)}$, $F^{\left(t,y,z+1\right)}$ and $F^{\left(t,y,z-1\right)}$ from the center panel, as well as the values Ω and Γ. For each step in time, the scheme will update the values in the right panel from the values of the center panel and write these values back to the center grip. By pressing F9 or performing any recorded input in Microsoft Excel an additional step in time will be performed. Below the center and the right panel are iteration counters that increment any time an input key or F9 (which triggers a spreadsheet recalculation) is recorded. By keeping F9 pressed, the evolution of the flow profile in time can be observed. Each step correlates to a step in time of $h_{t}$. An additional field is added to calculate the total number of microseconds passed since the beginning of the calculation.

### 3.4. Resetting the Calculation and Implementing the Boundary Conditions

Upon close inspection, the cells in the center panel do not simply copy the values from the right panel. They are linked by a conditional expression. If a certain field below the center panel (the field labeled “Reset”) is empty, the value from the right panel is copied. If the “Reset” field is not empty, the value from the left panel is copied. This effectively resets the calculation and also clears the iteration counters, which are implemented with a similar conditional copy operation. Writing any letter, value or number into the “Reset” field will thus reset the calculation and copy the initial conditions into the center panel corresponding to the velocity profile at t=0.

## 4. Analytical Solution for Initiating Two-Dimensional Flow in Rectangular Channel Cross-Sections

### 4.1. Derivation

In order to verify the correctness of the numerical results obtained from the implemented solver in Microsoft Excel, we chose the case of initiating two-dimensional flow in a rectangular channel cross-section with the no-slip boundary condition as an example. In this scenario, the flow in the channel is originally at rest. At t=0, a driving pressure gradient is applied along the length of the channel thus initiating the flow whereby the characteristic velocity profile in a rectangular channel cross-section Poiseuille flow will form. The dynamics of the evolution of this flow profile will be studied using the derived analytical solution which yields the exact results. These will then be compared to the numerical results obtained from the spreadsheet.

The relevant partial differential equation for this case is Equation (2) which is rewritten to

(10) $$\frac{\rho }{\eta }\frac{\partial v_{x}}{\partial t}-\left(\frac{\partial^{2}v_{x}}{\partial y^{2}}+\frac{\partial^{2}v_{x}}{\partial z^{2}}\right)=-\frac{\Delta p}{\eta \Delta L}$$

We assume the solution to consist of a steady-state component which is time-independent and a transient solution which is time-dependent. The latter will be dominating during the initiation of the flow and decline as the flow achieves steady-state. Thus, the general solution will be

(11) $$v_{x}\left(t,y,z\right)=v_{x,\mathrm{steady}-\mathrm{state}}\left(y,z\right)+v_{x,\mathrm{transient}}\left(t,y,z\right)$$

If inserted into Equation (10) we obtain

(12) $$\begin{array}{ll} \frac{\rho }{\eta }\left(\frac{v_{x,\mathrm{transient}}}{\partial t}\right)- & \left(\frac{\partial^{2}v_{x,\mathrm{steady}-\mathrm{state}}}{\partial y^{2}}+\frac{\partial^{2}v_{x,\mathrm{steady}-\mathrm{state}}}{\partial z^{2}}+\frac{\partial^{2}v_{x,\mathrm{transient}}}{\partial y^{2}}+\frac{\partial^{2}v_{x,\mathrm{transient}}}{\partial z^{2}}\right)\\ & =-\frac{\Delta p}{\eta \Delta L} \end{array}$$

Please note that $\frac{\partial v_{x,\mathrm{steady}-\mathrm{state}}}{\partial t}=0$. The steady-state solution $v_{x,\mathrm{steady}-\mathrm{state}}$ for this flow case is known and was derived in a similar case in Richter et al. [7] as Equation 13’ using an *eigenvalue* expansion for mixed boundary cases. The solution for no-slip boundary conditions is derived elsewhere ([8], Equation 16.62)

(13) $$\begin{array}{c} v_{x,\mathrm{steady}-\mathrm{state}}\left(y,z\right)=-\frac{16}{\eta \cdot \pi^{2}}\frac{\Delta p}{\Delta L}\overset{\infty }{\underset{n=0}{\sum }}\overset{\infty }{\underset{m=0}{\sum }}\sin\left(\left(2n+1\right)\pi \frac{y}{W}\right)\sin\left(\left(2m+1\right)\pi \frac{z}{H}\right)\\ {\left(\left(2n+1\right)\left(2m+1\right)\left({\left(\frac{\left(2n+1\right)\pi }{W}\right)}^{2}+{\left(\frac{\left(2m+1\right)\pi }{H}\right)}^{2}\right)\right)}^{-1} \end{array}$$

The second-order partial differentials required for Equation (12) are given by

(14) $$\begin{array}{c} \frac{\partial^{2}v_{x,\mathrm{steady}-\mathrm{state}}}{\partial y^{2}}=\frac{16}{\eta \cdot \pi^{2}}\frac{\Delta p}{\Delta L}\overset{\infty }{\underset{n=0}{\sum }}\overset{\infty }{\underset{m=0}{\sum }}{\left(\frac{\left(2n+1\right)\pi }{W}\right)}^{2}\sin\left(\left(2n+1\right)\pi \frac{y}{W}\right)\sin\left(\left(2m+1\right)\pi \frac{z}{H}\right)\\ {\left(\left(2n+1\right)\left(2m+1\right)\left({\left(\frac{\left(2n+1\right)\pi }{W}\right)}^{2}+{\left(\frac{\left(2m+1\right)\pi }{H}\right)}^{2}\right)\right)}^{-1} \end{array}$$

(15) $$\begin{array}{c} \frac{\partial^{2}v_{x,\mathrm{steady}-\mathrm{state}}}{\partial z^{2}}=\frac{16}{\eta \cdot \pi^{2}}\frac{\Delta p}{\Delta L}\overset{\infty }{\underset{n=0}{\sum }}\overset{\infty }{\underset{m=0}{\sum }}{\left(\frac{\left(2m+1\right)\pi }{H}\right)}^{2}\sin\left(\left(2n+1\right)\pi \frac{y}{W}\right)\sin\left(\left(2m+1\right)\pi \frac{z}{H}\right)\\ {\left(\left(2n+1\right)\left(2m+1\right)\left({\left(\frac{\left(2n+1\right)\pi }{W}\right)}^{2}+{\left(\frac{\left(2m+1\right)\pi }{H}\right)}^{2}\right)\right)}^{-1} \end{array}$$

(16) $$\begin{array}{l} \frac{\partial^{2}v_{x,\mathrm{steady}-\mathrm{state}}}{\partial y^{2}}+\frac{\partial^{2}v_{x,\mathrm{steady}-\mathrm{state}}}{\partial z^{2}}=\frac{16}{\eta \cdot \pi^{2}}\frac{\Delta p}{\Delta L}\\ \;\overset{\infty }{\underset{n=0}{\sum }}\overset{\infty }{\underset{m=0}{\sum }}\sin\left(\left(2n+1\right)\pi \frac{y}{W}\right)\sin\left(\left(2m+1\right)\pi \frac{z}{H}\right){\left(\left(2n+1\right)\left(2m+1\right)\right)}^{-1} \end{array}$$

Equation (16) is the representation of a constant by a two-dimensional Fourier series. Details on this can be found elsewhere ([8], Equation 4.44). We can thus simplify Equation (16) to

(17) $$\frac{\partial^{2}v_{x,\mathrm{steady}-\mathrm{state}}}{\partial y^{2}}+\frac{\partial^{2}v_{x,\mathrm{steady}-\mathrm{state}}}{\partial z^{2}}=\frac{\Delta p}{\eta \Delta L}$$

Using Equation (17), we can rewrite Equation (12) to

(18) $$\frac{\rho }{\eta }\frac{v_{x,\mathrm{transient}}}{\partial t}-\frac{\partial^{2}v_{x,\mathrm{transient}}}{\partial y^{2}}-\frac{\partial^{2}v_{x,\mathrm{transient}}}{\partial z^{2}}=0$$

Equation (18) is a homogeneous partial differential equation which can be solved by a separation of variables approach using

(19) $$v_{x,\mathrm{transient}}=T\left(t\right)Y\left(y\right)Z\left(z\right)$$

Inserting Equation (19) into Equation (18) yields

(20) $$\frac{\rho }{\eta }\frac{1}{T}\frac{\mathrm{dT}}{dt}-\frac{1}{Y}\frac{d^{2}Y}{dy^{2}}-\frac{1}{Z}\frac{d^{2}Z}{dz^{2}}=0$$

from which we derive the two second-order ordinary differential equations

(21) $$\frac{1}{Y}\frac{d^{2}Y}{dy^{2}}=-\lambda_{Y}^{2}$$

(22) $$\frac{1}{Z}\frac{d^{2}Z}{dz^{2}}=-\lambda_{Z}^{2}$$

and one first-order differential equation

(23) $$\frac{\rho }{\eta }\frac{1}{T}\frac{\mathrm{dT}}{dt}=-\left(\lambda_{Y}^{2}+\lambda_{Z}^{2}\right)$$

The solution to Equation (23) is straight-forward and given by integration as

(24) $$T\left(t\right)=C_{0}\;e^{-\left(\lambda_{Y}^{2}+\lambda_{Z}^{2}\right)\frac{\eta }{\rho }\;t}$$

The solutions to Equations (21) and (22) are given by the *eigenfunctions* and derived in a similar fashion as shown in [7] to be

(25) $$Y\left(y\right)=\sum_{n=0}^{\infty }C_{n}\sin\left(n\pi \frac{y}{W}\right),\;\lambda_{Y}=\frac{n\pi }{W}$$

(26) $$Z\left(z\right)=\sum_{m=0}^{\infty }C_{m}\sin\left(m\pi \frac{z}{H}\right),\;\lambda_{Z}=\frac{m\pi }{H}$$

Inserting Equations (24), (25) and (26) into Equation (19) yields the transient solution as

(27) $$v_{x,\mathrm{transient}}\left(t,y,z\right)=e^{-\left(\lambda_{Y}^{2}+\lambda_{Z}^{2}\right)\frac{\eta }{\rho }\;t}\overset{\infty }{\underset{n=0}{\sum }}\overset{\infty }{\underset{m=0}{\sum }}C_{nm}\sin\left(n\pi \frac{y}{W}\right)\sin\left(m\pi \frac{z}{H}\right)$$

We still lack the constant $C_{nm}$, which we can derive from the initial condition of the accelerating flow which requires the flow to be constant, i.e., $v_{x}=0$. This is the case for $v_{x,\mathrm{transient}}\left(t=0,y,z\right)=-v_{x,\mathrm{steady}-\mathrm{state}}\left(0,y,z\right)$. In this case we find

(28) $$\begin{array}{ll} \overset{\infty }{\underset{n=0}{\sum }}\overset{\infty }{\underset{m=0}{\sum }}C_{nm} & \sin(\left(2n+1\right)\pi \frac{y}{W})\sin\left(\left(2m+1\right)\pi \frac{z}{H}\right)\\ & =\frac{16}{\eta \cdot \pi^{2}}\frac{\Delta p}{\Delta L}\overset{\infty }{\underset{n=0}{\sum }}\overset{\infty }{\underset{m=0}{\sum }}\sin\left(\left(2n+1\right)\pi \frac{y}{W}\right)\sin\left(\left(2m+1\right)\pi \frac{z}{H}\right)\\ & {\left(\left(2n+1\right)\left(2m+1\right)\left({\left(\frac{\left(2n+1\right)\pi }{W}\right)}^{2}+{\left(\frac{\left(2m+1\right)\pi }{H}\right)}^{2}\right)\right)}^{-1} \end{array}$$

from which we find

(29) $$C_{nm}=\frac{16}{\eta \cdot \pi^{2}}\frac{\Delta p}{\Delta L}{\left(\left(2n+1\right)\left(2m+1\right)\left({\left(\frac{\left(2n+1\right)\pi }{W}\right)}^{2}+{\left(\frac{\left(2m+1\right)\pi }{H}\right)}^{2}\right)\right)}^{-1}$$

Assembling Equation (11) from Equations (13), (27) and (29) we find

(30) $$\begin{array}{ll} v_{x}\left(t,y,z\right)= & -\frac{16}{\eta \cdot \pi^{2}}\frac{\Delta p}{\Delta L}\overset{\infty }{\underset{n=0}{\sum }}\overset{\infty }{\underset{m=0}{\sum }}\begin{array}{c} \left(1-e^{-\left({\left(\frac{\left(2n+1\right)\pi }{W}\right)}^{2}+{\left(\frac{\left(2m+1\right)\pi }{H}\right)}^{2}\right)\;\frac{\eta }{\rho }\;t}\right)\\ \sin\left(\left(2n+1\right)\pi \frac{y}{W}\right)\sin\left(\left(2m+1\right)\pi \frac{z}{H}\right) \end{array}\\ & {\left(\left(2n+1\right)\left(2m+1\right)\left({\left(\frac{\left(2n+1\right)\pi }{W}\right)}^{2}+{\left(\frac{\left(2m+1\right)\pi }{H}\right)}^{2}\right)\right)}^{-1} \end{array}$$

#### Visualization

**Figure 2:**
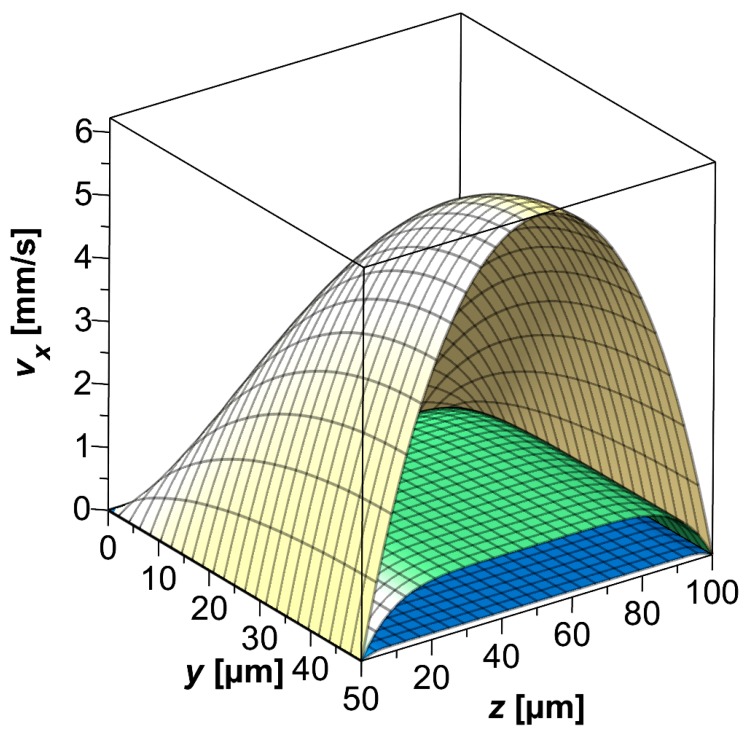
Velocity profile calculated from the analytical solution of Equation (30) for water as fluid and a channel with 100 μm width and 100 μm height. The Fourier series is expanded to $n_{\max}=m_{\max}=10$. The expansion order of the Fourier series was 10 both along y and z. Velocity profiles for different points in time are plotted: 10 μs (dark blue), 100 μs (medium green) and 1000 μs (light yellow).

In the following, Equation (30) is visualized using a microfluidic channel with 100 μm width and 100 μm height and choosing water (η=1 mPa·s, $\rho =1\;g/{\mathrm{cm}}^{3}$) as the fluid. The Fourier series in Equation (30) is expanded to $n_{\max}=m_{\max}=10$. Figure 2 shows the calculated profiles for 10 μs, 100 μs and 1000 μs. As can be seen the fluid is originally at rest. Over time, the characteristic profile of the Poiseuille flow in a rectangular channel cross-section evolves. By inspection of Equations (27) and (30), this behavior is expected. As the solution consists of a transient and a steady-state component, the first of which decays exponentially over time, the steady-state solution is effectively modulated by the exponential decay of the time-dependent contribution of the transient solution.

### 4.2. Application of the Derived Spreadsheet

#### 4.2.1. Initiating Two-Dimensional Flow in Rectangular Channel Cross-Sections

**Figure 3:**
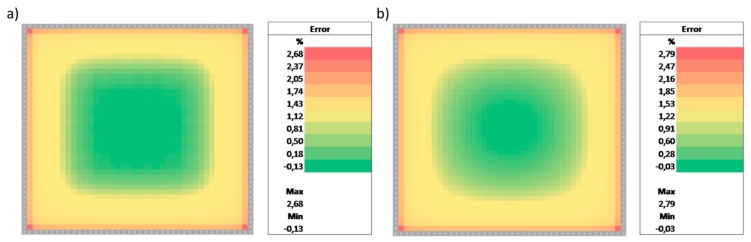
Comparison of the results obtained from the numerical scheme implemented in Microsoft Excel against the analytical solution of Equation (30) for time point 100 μs (**a**) and 1000 μs (**b**). The graphs show the relative error. Both cases show very good agreements with maximum errors below 3%.

Given the analytical solution Equation (30) we now proceed to solving the same case using the numerical solver implemented in the spreadsheet. The step width in space was chosen as $h_{yz}=2.5\;\mu m$ and the step width in time as $h_{t}=1\;\mu s$. Again, water as the fluid was assumed and thus η=1 mPa·s, $\rho =1\;g/{\mathrm{cm}}^{3}$ were set in the “Variables” section of the spreadsheet. For direct comparison with Figure 2, the spreadsheet was iterated 100 times to yield the velocity profile at t=100 μs and t=1000 μs. The resulting velocity profile was then compared with the analytical Page: 10 solution given by Equation (30). Figure 3 shows the relative error of the numerical output compared to the analytical solution. The maximum error in the whole computational domain is below 3% relative error in both cases. The errors are strongest in regions of steep gradients in the velocity profiles, i.e., at the edges. In order to decrease the error further, the computational domain can be increased by increasing the number of cells. However, even on this rather coarse and compact domain, the errors fall within acceptable limits.

#### 4.2.2. Complex Flow Cases: Different Channel Cross-Sections

**Figure 4:**
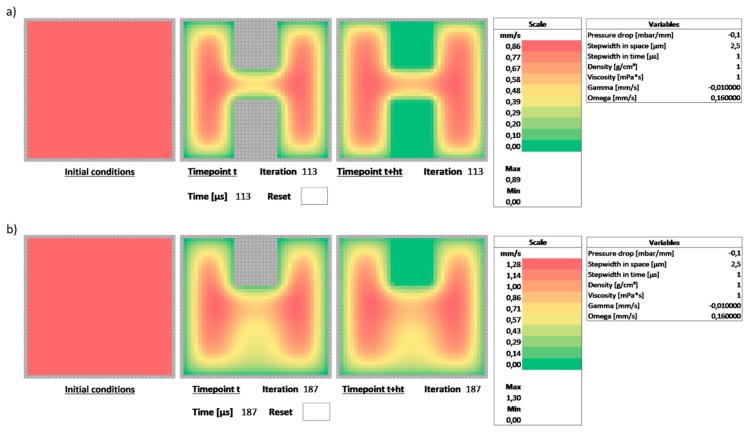
Complex flow cases involving intricate domains such as a double-constricted channel cross-section. (**a**) These are implemented by adding additional boundary values, i.e., by copying the grey cell values which have static values. The image shows the flow after around 100 iterations. (**b**) By simple overwriting portions of the boundary values with domain values, i.e., reimplementing the conditional copy operation of the center values, one of the obstacles is removed at a given point in time. The scheme can then be iterated to see how the flow adapts to the changes in boundary conditions.

As shown, the analytical solution can be derived for the (rather simplistic) case of rectangular channel cross-sections. However, the availability of these solutions is limited if the channel geometries become more complex. However, these cross-sections are straight-forward to implement in the spreadsheet. Figure 4a shows the example of a rectangular channel with two fins constricting the flow.

These can be implemented by copying additional boundary values into the center panel. By overwriting the formulae in these cells, the numerical scheme cannot iterate these values. However, they influence neighboring cells and thus show up as regions with no flow in the iterated panel on the right. Again, starting with a static flow, the scheme can be used to observe the development of the velocity profile over time. Figure 4a shows the profile at iteration #113, i.e., at t=113 μs. Boundary conditions can be changed dynamically. As an example, the lower constriction in the channel of Figure 4a was removed at t=113 μs. Figure 4b shows the adjusting flow 74 iterations after the object was removed. As can be seen, the flow re-expands into the regions blocked previously. Removing a boundary can be accomplished by simply recopying the formulae from the domain into the center panel thus reactivating the iteration.

#### 4.2.3. Boundary Conditions and Initial Conditions

As discussed, boundary conditions with fixed values (Dirichlet-type) can be implemented by overwriting cells in the center panel with fixed values. No-slip boundary conditions are implemented by setting this value to 0. Surfaces with fixed velocity as in the case of, e.g., Couette flow, can be implemented by setting the velocity of these boundary cells to a fixed non-zero value. As demonstrated in [7], it is possible to also implement Neumann-type boundary conditions by simply setting the cell values of the boundary to the value of the adjacent cell within the computational domain thus effectively generating a velocity gradient of 0 as required for, e.g., slip boundary conditions. Obviously, the gradient can also be set to a fixed value by simply setting the value of the boundary cell to the value of the neighboring cell within the computational domain plus a fixed offset value.

**Figure 5:**
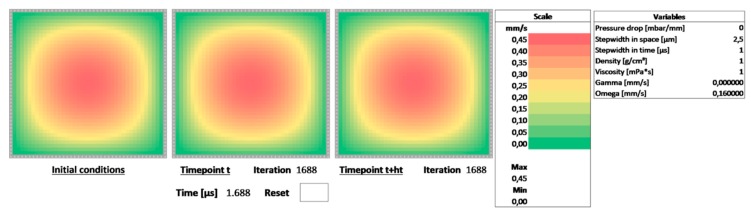
Example of a flow case using boundary conditions. The demonstrated case is a decelerating flow which was initially fully-defined as indicated by the initial conditions. At t=0 the driving pressure gradient is removed and the flow begins to stagnate. After more than 1500 iterations, the velocity profile is smeared and the velocity profile drops to maximum values below 0.5 mm/s.

It is also possible to use non-zero initial values for computation. Until now, we assumed the flow to be static at the beginning of the experiment and to increase due to the application of the flow as a consequence of the application of a pressure gradient. This spreadsheet analyses the declining velocity profile of an initially moving fluid as a consequence of the removal of the driving pressure. The initial values are copied from a spreadsheet that iterated until the velocity profile was fully developed. The spreadsheet of Figure 5 sets the driving pressure gradient to 0. As you can see, after 1000 iterations the velocity profile has smeared slightly but the overall distribution is still similar to the case of the fully-developed profile. This is to be expected from the analytical solution to Equation (27). For a decelerating flow, the steady-state fully-developed flow profile would be modulated by an exponential term, thus generating an exponential decay. As a consequence, the flow profiles would simply be scaled but the shape of the profile would remain largely intact. As can be seen from Figure 5, after 1000 iterations, the maximum velocity in the channel has decreased to a value of below 0.5 mm/s.

## 5. Conclusions

Solutions to the simplified Navier–Stokes equation in pressure-driven microfluidics, i.e., Poiseuille flows, are difficult to derive analytically if the channel cross-sections, the boundary conditions or the initial values do not represent trivial cases. Including time-dependency further complicates the derivation of the analytical solutions. These cases are usually addressed using numerical solvers and dedicated software packages. However, as we have shown, a numerical solver suitable for solving time-dependent microfluidic flow cases in arbitrary channel cross-sections can be conveniently implemented using spreadsheet analysis tools such as, e.g., Microsoft Excel. By making use of a controlled iterative calculation, the solver can be stepped in time by manual input thus allowing a precise study of the evolving velocity profiles over time at discrete time points. No additional software is required for obtaining results that almost exactly correspond to the precise analytical solution. We demonstrated this using the case of time-dependent initiating flow in a rectangular channel cross-section. The spreadsheet developed can be used to implement almost any type of boundary condition or initial condition, as well as channel cross-section as required. We believe that providing researchers with intuitive and widely accessible numerical tools will significantly increase the understanding and the correct derivation of the fluid mechanics in microfluidics with implications for applications in liquid delivery, reaction synthesis and analytical applications [9,10,11]. Spreadsheet software packages such as, e.g., Microsoft Excel are widely available and most scientists are used to working with these tools in routine lab work. Using these tools effectively to provide such detailed insight into fluid mechanics will significantly widen the application range and provide more detailed understanding of phenomena which are generally only accessible with specialized software packages.

## Supplementary Materials

The following are available online at https://www.mdpi.com/2079-6374/9/2/67/s1, Microsoft Excel spreadsheet “TimedependentMicrofluidicFlows.xlsx”.
